# Supplementary material for: A comparative study of compartmental models for COVID-19 transmission in Ontario, Canada
Source: Sci Rep. 2023 Sep 12;13:15050. doi: 10.1038/s41598-023-42043-y (PMC10497623; doi:10.1038/s41598-023-42043-y)
Supplement: Supplementary file 1 — Supplementary Information. [file 41598_2023_42043_MOESM1_ESM.pdf]

Supplementary Information

A. $SV^2(AIR)^3$ model

Following Layton and Sadria [4], the model is initialized on January 1, 2020, and simulates the emergence of wild-type, Alpha-type, Delta-type until Fall 2021. From November 22, 2021 onwards, a new variant (which we have updated to mimic Omicron-type characteristics) replaces wild-type. We extracted the model output for our investigated period of January 6, 2022 to June 4, 2022.

Table S1: Parameters in the $SV^2(AIR)^3$ model for the wild-type, alpha-type and Delta-type variants as provided in Layton and Sadria [4]. Definitions of each model parameter are provided below, and a full description of the model can be found in Layton and Sadria [4].

Parameters Included	Variant of Concern(X)		
	X=Wild	X=Alpha	X=Delta
β^X [1]	0.0481	0.0801	0.1107
β^X_{V1} [2]	0.0096	0.0401	0.0742
$\beta^X_{V2=PZ}$ [3]	0.0024	0.0056	0.0133
$\beta^X_{V2=AZ}$ [4]	0.0024	0.0272	0.0433
β^X_R [5]	0.0024	0.0040	0.0055
α^X [6]	3	3	3
η^X_{V1} [7]	$2 \cdot 0.25/182$	$2 \cdot 0.25/182$	$2 \cdot 0.25/182$
$\eta^X_{V2=PZ,AZ}$ [8]	$2 \cdot 0.125/365$	$2 \cdot 0.125/365$	$2 \cdot 0.125/365$
η_R [9]	$2 \cdot 0.125/365$	$2 \cdot 0.125/365$	$2 \cdot 0.125/365$
μ [10]	0.00002	0.00002	0.00002
μ^X [11]	0.0010	0.0017	0.0019
μ^X_V [12]	0.00015	0.000255	0.000285
γ^X [13]	1/28	1/28	1/28
σ^X [14]	0.5	0.5	0.5
σ^X_V [15]	0.85	0.85	0.85
σ^X_R [16]	0.85	0.85	0.85

Notes:
[1] Disease transmission rate without vaccination
[2] Disease transmission rate after partial vaccination
[3] Disease transmission rate after taking full dose of Pfizer-BioNTech or Moderna
[4] Disease transmission rate after taking full dose of Astra-Zeneca
[5] Disease re-infection rate
[6] Ratio between asymptomatic and symptomatic infectivity
[7] Loss of immunity rate after partial vaccination
[8] Loss of immunity rate after full vaccination
[9] Loss of immunity rate after recovering from previous infection
[10] Natural death rate
[11] Disease mortality rate without vaccination
[12] Disease mortality rate after vaccination
[13] Disease recovery rate
[14] Fraction of asymptomatic infections without vaccination
[15] Fraction of asymptomatic infections after vaccination
[16] Fraction of asymptomatic infections after recovering from previous infection

Table S2: Parameters in the SV^2(AIR)^3 model for the newly-emerging variant [4] and our updated values for the Omicron variant. Parameter definitions are the same as in Table S1.

Parameters Included	Variant of Concern(X)	
	X= Omicron	X= Hypothetical Variant
β^X	0.5000	0.1262
β^X_{V1}	0.4250	0.0883
$\beta^X_{V2=PZ}$	0.3500	0.0315
$\beta^X_{V2=AZ}$	0.3500	0.0631
β^X_R	0.0250	0.0063
α^X	3	3
η^X_{V1}	$2 \cdot 0.25/182$	$2 \cdot 0.25/182$
$\eta^X_{V2=PZ,AZ}$	$2 \cdot 0.125/365$	$2 \cdot 0.125/365$
η_R	$2 \cdot 0.125/365$	$2 \cdot 0.125/365$
μ	0.00002	0.00002
μ^X	0.0019	0.0010
μ^X_V	0.000284	0.00015
γ^X	1/8	1/28
σ^X	0.6	0.55
σ^X_V	0.85	0.85
σ^X_R	0.85	0.85

B. Vaccination-stratified SEPAIQRD model

B.1 Plot of daily confirmed cases after data processing step

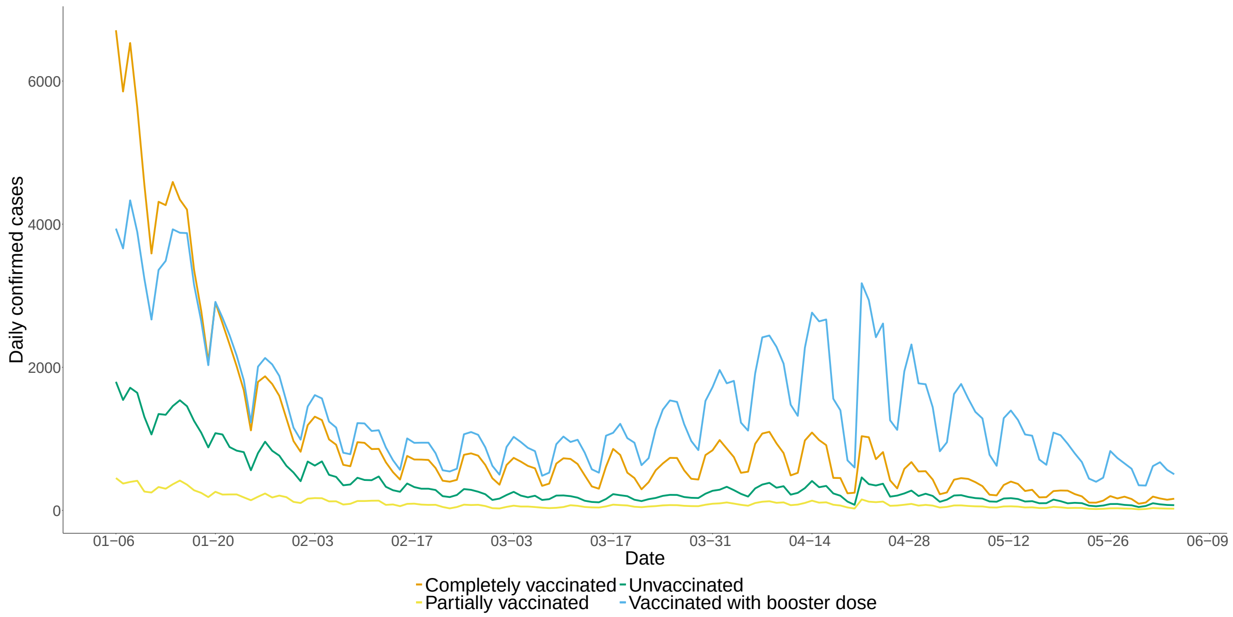

Figure S1: Daily confirmed COVID-19 cases from January 6, 2022 to June 4, 2022 in Ontario stratified by four vaccination statuses: unvaccinated, partially vaccinated, completely vaccinated, and vaccinated with booster dose. From January 6 to March 10, we split Ontario's 'fully vaccinated' infections according to the daily proportion of completely vaccinated and vaccinated with booster dose populations in Ontario. After March 10, we split the 'not fully vaccinated' infections according to the daily proportion of unvaccinated and partially vaccinated populations in Ontario.

B.2 Detailed description of the disease transmission stage

In the disease transmission stage, the interactions within the same group and between different groups are considered. We follow the idea from existing literature [3] to quantify the transmission rate between stratified groups using Equation S1:

$$\beta_{ij} = c_{ij} \cdot p, \tag{S1}$$

where p is defined as the transmission probability per contagious contact and c_{ij} is defined as the daily contact rate between the ith and jth groups. To the best of our knowledge, there are not any available data evaluating the contact rate between groups with different vaccination statuses. We take the idea of the contact matrix given age from Fields et al. [3], and apply it to construct a contact matrix given vaccination status assuming independence. The transformed contact matrix is shown in Table S3. Then, we set $p = 0.02$ according to Syga et al. [5].

The individuals who are considered contagious include those in the documented symptomatic infectious compartment (I_i), symptomatic infectious compartment (P_i), asymptomatic infectious compartment (A_i), and two transition compartments (T_i and T_i'). Different compartments may transmit the disease at different rates: we let $\beta_{I,ij}, \beta_{P,ij}, \beta_{A,ij}, \beta_{T,ij}, \beta_{T',ij}$, respectively denote the

Table S3: Contact matrix given groups with different vaccination statuses. Note that $c_{ij} \neq c_{ji}$ since the case where people in the ith group actively contact people in the jth group is distinct from the case where people in the jth group actively contact people in the ith group.

		Passive Contact Group(j)			
		Unvaccinated	Partially vaccinated	Completely vaccinated	Vaccinated with booster
Active Contact Group(i)	Unvaccinated	2.4844	1.1046	6.3798	4.1864
	Partially vaccinated	2.7819	1.3353	6.3711	3.1741
	Completely vaccinated	1.8744	0.7432	5.7009	5.3397
	Vaccinated with booster	0.9392	0.2827	4.0765	6.3317

transmission rates of these contagious compartments. We assume people in P_i, T_i, and T_i' are as contagious as those in I^i, such that

$$\beta_{P,ij} = \beta_{T,ij} = \beta_{T',ij} = \beta_{I,ij} = \beta_{ij} = c_{ij} \cdot p. \tag{S2}$$

We also assume those in A_i are not as contagious as those in I_i, such that

$$\beta_{A_ij} = 0.2 \cdot \beta_{ij} = 0.2 \cdot c_{ij} \cdot p. \tag{S3}$$

With the setup above, we can compute the disease transmission rates of I_i, P_i and A_i using Equations S2 and S3. Table S4 shows the disease transmission rates of the I_i and P_i compartments, while Table S5 shows the disease transmission rate of compartment A_i.

Table S4: Disease transmission rates of the I_i, P_i, T_i, and T_i' compartments, which are respectively denoted as $\beta_{I,ij}$, $\beta_{P,ij}$, $\beta_{T,ij}$, and $\beta_{T',ij}$

		Passive Contact Group(j)			
		Unvaccinated	Partially vaccinated	Completely vaccinated	Vaccinated with booster
Active Contact Group(i)	Unvaccinated	0.0497	0.0221	0.1276	0.0837
	Partially vaccinated	0.0556	0.0267	0.1267	0.0635
	Completely vaccinated	0.0375	0.0149	0.1140	0.1068
	Vaccinated with booster	0.0188	0.0057	0.0815	0.1266

Table S5: Disease transmission rate of the A_i compartment, which is denoted as $\beta_{A,ij}$

		Passive Contact Group(j)			
		Unvaccinated	Partially vaccinated	Completely vaccinated	Vaccinated with booster
Active Contact Group(i)	Unvaccinated	0.0099	0.0044	0.0255	0.0167
	Partially vaccinated	0.0111	0.0053	0.0253	0.0127
	Completely vaccinated	0.0750	0.0030	0.0228	0.0214
	Vaccinated with booster	0.0038	0.0011	0.0163	0.0253

The impact of policy changes on COVID-19 transmission is also considered. We use the time-varying Oxford Stringency Index to quantify this impact. We scale our β_{ij} by the time-dependent $1-\lambda(t)$. This is a piece-wise constant function, which means that it will remain constant within the same reopening phase and changes as the Ontario government moves from one phase to another. The changepoints used in the definition of $1-\lambda(t)$ align with the changes in reopening states described in the Data Description section of the main paper.

B.3 Fixed model parameters

Estimates of the case fatality proportion by vaccination status are obtained from a long-term observational study [2], which need to be converted to an equivalent death rate (with the unit of days^{-1}) for use in our model.

Let ζ_i denote the case fatality proportion for vaccination status i. The total flow out from the I_i compartment is the sum of the flows $I_i \to Q_i$, $I_i \to T_i$, and $I_i \to D_i$. At the model's steady state, the case fatality proportion is therefore the proportion of that total represented by the $I_i \to D_i$ flow, namely

$$\zeta_i = \frac{\alpha_i}{\alpha_i + (1-\alpha_i)(1-\epsilon)\kappa_{I\to Q} + (1-\alpha_i)(\epsilon)\kappa_{I\to Q}}, \tag{S4}$$

where α_i represents the implied death rate (in days^{-1}). Since the self-isolation delay $\kappa_{I\to Q}$ is set to 1 day, Equation S4 simplifies to $\zeta_i = \alpha_i$. Consequently, the death rate α_i in our model is equal to the case fatality proportion ζ_i.

Sources for the other parameters are indicated in Table 2 of the main text.

Table S6: Values of fixed parameters in the vaccination-stratified SEPAIQRD model.

Model Parameter	Value
κ_E [1]	1/1.42 days^{-1}
κ_A	1/8.29 days^{-1}
$\kappa_{I\to Q}$	1 days^{-1}
$\kappa_{Q\to R}$	1/5.87 days^{-1}
$\kappa_{I\to R}$	1/6.87 days^{-1}
κ_P	1/2 days^{-1}
$\kappa_{P\to R'}$	1/8.29 days^{-1}
ϵ	0.96
α_1	0.3/100 days^{-1}
α_2 [2]	0.19/100 days^{-1}
α_3	0.08/100 days^{-1}
α_4	0.07/100 days^{-1}

Notes:
[1] The incubation period, which consists of a 2-day pre-symptomatic infectious period [1] and a latent period, is 3.42 days [6]. Together, this implies a latent period of 1.42 days.
[2] We approximate this value by averaging the death rates of the unvaccinated and fully vaccinated groups, since the cited study did not have sufficient data to provide a separate estimate for the partially vaccinated group.

B.4 System of differential equations

This section provides the system of differential equations that governs the dynamic mechanisms of our vaccination-stratified SEPAIQRD model, corresponding to the schematic in Figure 2 of the main text.

We define N_1, N_2, N_3, N_4 to be piecewise-constant functions varying by day, with values corresponding to the sub-population sizes of the four vaccination statuses. Likewise, we define V_1, V_2, V_3 to be piecewise-constant functions varying by day, which are given by the number of first, second, and third doses given to individuals per day. Both N_i and V_j are directly calculated from the COVID-19 vaccination data from Public Health Ontario. Then, the differential equations at the disease transmission stage for the four susceptible subpopulations are governed by Equations S5, S6, S7, and S8 as follows:

$$\frac{\mathrm{d}S_1}{\mathrm{d}t} = -\sum_{j=1}^{4} \frac{S_1}{N_j} \left(\beta_{P,1j}P_j + \beta_{I,1j}I_j + \beta_{A,1j}A_j + \beta_{T,1j}T^j + \beta_{T',1j}T'_j\right) \cdot (1-\lambda(t)) - V_1 \tag{S5}$$

$$\frac{\mathrm{d}S_2}{\mathrm{d}t} = -\sum_{j=1}^{4} \frac{S_2}{N_j} \left(\beta_{P,2j}P_j + \beta_{I,2j}I_j + \beta_{A,2j}A_j + \beta_{T,2j}T_j + \beta_{T',2j}T'_j\right) \cdot (1-\lambda(t)) + V_1 - V_2 \tag{S6}$$

$$\frac{\mathrm{d}S_3}{\mathrm{d}t} = -\sum_{j=1}^{4} \frac{S_3}{N_j} \left(\beta_{P,3j}P_j + \beta_{I,3j}I_j + \beta_{A,3j}A_j + \beta_{T,3j}T_j + \beta_{T',3j}T'_j\right) \cdot (1-\lambda(t)) + V_2 - V_3 \tag{S7}$$

$$\frac{\mathrm{d}S_4}{\mathrm{d}t} = -\sum_{j=1}^{4} \frac{S_4}{N_j} \left(\beta_{P,4j}P_j + \beta_{I,4j}I_j + \beta_{A,4j}A_j + \beta_{T,4j}T_j + \beta_{T',4j}T'_j\right) \cdot (1-\lambda(t)) + V_3. \tag{S8}$$

The remaining compartments are governed by Equations S9–S19:

$$\frac{\mathrm{d}E_i}{\mathrm{d}t} = \sum_{j=1}^{4} \frac{S_i}{N_j} \left(\beta_{P,ij}P_j + \beta_{I,ij}I_j + \beta_{A,ij}A_j + \beta_{T,ij}T_j + \beta_{T',ij}T'_j\right) \cdot (1-\lambda(t)) - (1-f_i(t)) \cdot \kappa_E \cdot E_i - f_i(t) \cdot \kappa_E \cdot E_i \tag{S9}$$

$$\frac{\mathrm{d}A_i}{\mathrm{d}t} = f_i(t) \cdot \kappa_E \cdot E_i - \kappa_A A_i \tag{S10}$$

$$\frac{\mathrm{d}RA_i}{\mathrm{d}t} = \kappa_A A_i \tag{S11}$$

$$\frac{\mathrm{d}P_i}{\mathrm{d}t} = (1-f_i(t)) \cdot \kappa_E \cdot E_i - \kappa_P \cdot CAR(t) \cdot P_i - \kappa_P \cdot (1-CAR(t)) \cdot P_i \tag{S12}$$

$$\frac{\mathrm{d}T'_i}{\mathrm{d}t} = (1-CAR(t)) \cdot \kappa_P \cdot P_i - \frac{1}{\frac{1}{\kappa_{P\to R'}} - \frac{1}{\kappa_P}} \cdot T'_i \tag{S13}$$

$$\frac{\mathrm{d}R'_i}{\mathrm{d}t} = \frac{1}{\frac{1}{\kappa_{P\to R'}} - \frac{1}{\kappa_P}} \cdot T'_i \tag{S14}$$

$$\frac{\mathrm{d}I_i}{\mathrm{d}t} = \kappa_P \cdot CAR(t) \cdot P_i - (\alpha_i + (1-\epsilon) \cdot (1-\alpha_i) \cdot \kappa_{I\to R} + (1-\alpha_i) \cdot \epsilon \cdot \kappa_{I\to Q}) \cdot I_i \tag{S15}$$

$$\frac{\mathrm{d}T^i}{\mathrm{d}t} = (1-\alpha_i) \cdot (1-\epsilon) \cdot \kappa_{I\to Q} \cdot I_i - \frac{1}{\frac{1}{\kappa_{I\to R}} - \frac{1}{\kappa_{I\to Q}}} \cdot T_i \tag{S16}$$

$$\frac{\mathrm{d}R_i}{\mathrm{d}t} = \frac{1}{\frac{1}{\kappa_{I\to R}} - \frac{1}{\kappa_{I\to Q}}} \cdot T_i + \frac{1}{\frac{1}{\kappa_{I\to R}} - \frac{1}{\kappa_{I\to Q}}} \cdot Q_i \tag{S17}$$

$$\frac{\mathrm{d}Q_i}{\mathrm{d}t} = (\kappa_{I\to Q} \cdot \epsilon \cdot (1 - \alpha_i))I_i - \frac{1}{\frac{1}{\kappa_{I\to R}} - \frac{1}{\kappa_{I\to Q}}} \cdot Q_i \tag{S18}$$

$$\frac{\mathrm{d}D_i}{\mathrm{d}t} = \alpha_i \cdot I_i \tag{S19}$$

B.5 Prior distributions of unknown parameters

Table S7: The prior distributions of the unknown parameters $\mathcal{L}(f_{i,j})$, $\mathcal{L}(CAR_j)$, and $(\phi_{i,j})^{-1}$. The subscript i indicates the vaccination status, and the subscript j indicates the reopening phase. As described in the main text, the prior means for $\mathcal{L}(CAR_j)$ are set to increase as Ontario moves through reopening phases. The prior means for the logit-transformed asymptomatic infection proportion are set by vaccination status and reopening phase, such that vaccination reduces symptoms (more likely to be asymptomatic with more doses) and efficacy of vaccination decays over the investigated period. Large prior standard deviations are set so that the posterior distributions will be primarily informed by the data.

Parameter	Prior distribution
$\mathcal{L}(f_{1,1})$	Normal(-1,2)
$\mathcal{L}(f_{2,1})$	Normal(-0.5,2)
$\mathcal{L}(f_{3,1})$	Normal(1.5,2)
$\mathcal{L}(f_{4,1})$	Normal(2,2)
$\mathcal{L}(f_{1,2})$	Normal(-1,2)
$\mathcal{L}(f_{2,2})$	Normal(-0.65,2)
$\mathcal{L}(f_{3,2})$	Normal(1.25,2)
$\mathcal{L}(f_{4,2})$	Normal(1.75,2)
$\mathcal{L}(f_{1,3})$	Normal(-1,2)
$\mathcal{L}(f_{2,3})$	Normal(-0.75,2)
$\mathcal{L}(f_{3,3})$	Normal(1,2)
$\mathcal{L}(f_{4,3})$	Normal(1.5,2)
$\mathcal{L}(f_{1,4})$	Normal(-1,2)
$\mathcal{L}(f_{2,4})$	Normal(-0.85,2)
$\mathcal{L}(f_{3,4})$	Normal(0.8,2)
$\mathcal{L}(f_{4,4})$	Normal(1.25,2)
$\mathcal{L}(f_{1,5})$	Normal(-1,2)
$\mathcal{L}(f_{2,5})$	Normal(-0.95,2)
$\mathcal{L}(f_{3,5})$	Normal(0.75,2)
$\mathcal{L}(f_{4,5})$	Normal(1.15,2)
$\mathcal{L}(CAR_1)$	Normal(-1.84,1.31)
$\mathcal{L}(CAR_2)$	Normal(-1.8, 1.15)
$\mathcal{L}(CAR_3)$	Normal(-1.5,1.31)
$\mathcal{L}(CAR_4)$	Normal(-1.09,1.15)
$\mathcal{L}(CAR_5)$	Normal(-0.59,1.31)
$(\phi_{i,j})^{-1}$	Exponential(5)

B.6 Posterior distributions of unknown parameters

Table S8: Summary of posterior distributions of CAR_j and $f_{i,j}$ on the original [0,1] scale. For each parameter, the posterior mean, lower quantile (0.025) of the 95% credible interval, and upper quantile (0.975) of the 95% credible interval are shown, based on the MCMC samples.

	Mean	Lower Quantile	Upper Quantile
$f_{1,1}$	0.0101	0.0010	0.0505
$f_{2,1}$	0.4543	0.3858	0.5171
$f_{3,1}$	0.1221	0.0485	0.2042
$f_{4,1}$	0.0278	0.0046	0.0890
$f_{1,2}$	0.0143	0.0012	0.0737
$f_{2,2}$	0.3323	0.2137	0.4360
$f_{3,2}$	0.3451	0.2407	0.4413
$f_{4,2}$	0.2652	0.1608	0.3657
$f_{1,3}$	0.0473	0.0035	0.1960
$f_{2,3}$	0.3992	0.2312	0.5520
$f_{3,3}$	0.2208	0.0712	0.3831
$f_{4,3}$	0.1627	0.0414	0.3260
$f_{1,4}$	0.2677	0.1579	0.3736
$f_{2,4}$	0.3740	0.2555	0.4852
$f_{3,4}$	0.2650	0.1588	0.3775
$f_{4,4}$	0.1286	0.0372	0.2443
$f_{1,5}$	0.2758	0.1929	0.3556
$f_{2,5}$	0.2091	0.1207	0.2993
$f_{3,5}$	0.4983	0.4345	0.5556
$f_{4,5}$	0.0406	0.0069	0.1230
CAR_1	0.1885	0.1708	0.2075
CAR_2	0.1815	0.1523	0.2117
CAR_3	0.2049	0.1642	0.2509
CAR_4	0.2110	0.1612	0.2570
CAR_5	0.4303	0.3350	0.5011

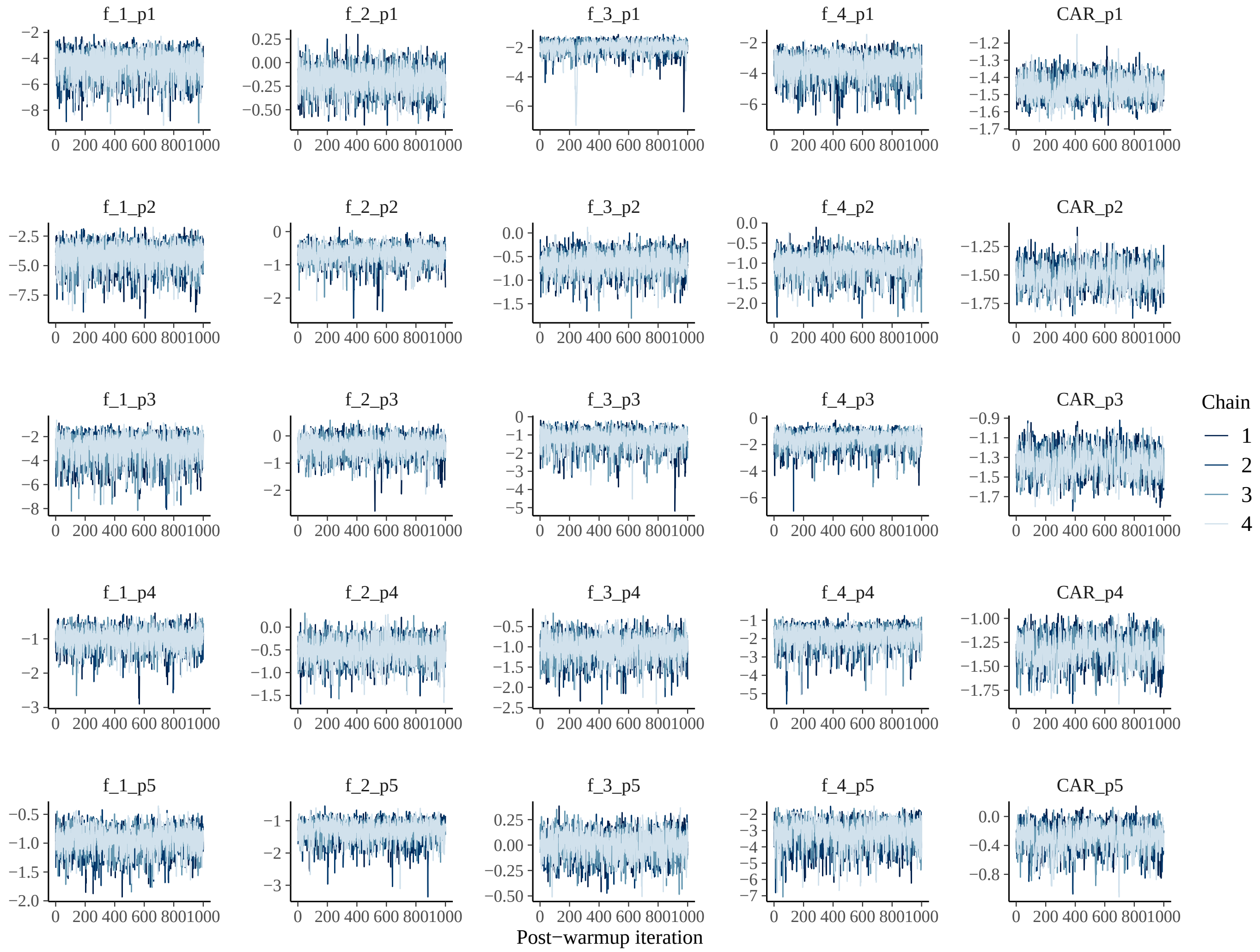

Figure S2: Traceplots of the MCMC samples for the model parameters $\mathcal{L}(f_{i,j})$, $\mathcal{L}(CAR_j)$, and $(\phi_i^j)^{-1}$.

References

[1] Sam Li-Sheng Chen, Grace Hsiao-Hsuan Jen, Chen-Yang Hsu, Amy Ming-Fang Yen, Chao-Chih Lai, Yen-Po Yeh, and Tony Hsiu-Hsi Chen. A new approach to modeling pre-symptomatic incidence and transmission time of imported COVID-19 cases evolving with SARS-CoV-2 variants. *Stochastic Environmental Research and Risk Assessment*, 37(1):441–452, 2023.

[2] Phoebe Danza, Tae Hee Koo, Meredith Haddix, Rebecca Fisher, Elizabeth Traub, Kelsey OYong, and Sharon Balter. SARS-CoV-2 infection and hospitalization among adults aged 18 years, by vaccination status, before and during SARS-CoV-2 B. 1.1. 529 (Omicron) variant predominance—Los Angeles County, California, November 7, 2021–January 8, 2022. *Morbidity and Mortality Weekly Report*, 71(5):177, 2022.

[3] R. Fields, L. Humphrey, D. Flynn-Primrose, Z. Mohammadi, M. Nahirniak, E.W. Thommes, and M.G. Cojocaru. Age-stratified transmission model of COVID-19 in Ontario with human mobility during pandemic's first wave. *Heliyon*, 7(9):e07905, 2021. ISSN 2405-8440. doi: https://doi.org/10.1016/j.heliyon.2021.e07905.

[4] Anita T Layton and Mehrshad Sadria. Understanding the dynamics of SARS-CoV-2 variants of concern in Ontario, Canada: A modeling study. *Scientific reports*, 12:2114, 2022.

[5] Simon Syga, Diana David-Rus, Yannik Schälte, Haralampos Hatzikirou, and Andreas Deutsch. Inferring the effect of interventions on COVID-19 transmission networks. *Scientific reports*, 11 (1):21913, 2021.

[6] Yu Wu, Liangyu Kang, Zirui Guo, Jue Liu, Min Liu, and Wannian Liang. Incubation period of COVID-19 caused by unique SARS-CoV-2 strains: a systematic review and meta-analysis. *JAMA network open*, 5(8):e2228008–e2228008, 2022.
